# Case of Severe Hiccups Causing SIADH, Treated With Lorazepam

**DOI:** 10.1002/ccr3.71048

**Published:** 2025-09-29

**Authors:** Lisa Matlen

**Affiliations:** ^1^ Pediatric Neurology, Department of Pediatrics, C.S. Mott Children's Hospital University of Michigan Ann Arbor Michigan USA

**Keywords:** case report, hiccups, hyponatremia, SIADH

## Abstract

This is a presentation of severe hiccups which led to the syndrome of inappropriate antidiuretic hormone (SIADH). The patient presented after 3 days of severe hiccups associated with vomiting and oral intake intolerance. SIADH was diagnosed by hyponatremia (initial 128 millimoles/liter (mmol/L), nadir 119 mmol/L; normal range 136–146 mmol/L) and urine studies; this was initially attributed to sertraline, but a 48‐h medication hold did not improve sodium and caused withdrawal. An upper gastrointestinal (GI) study demonstrated mesenteroaxial gastric malrotation with hiatal hernia; these were non‐operative. Hyponatremia was corrected with 3% sodium, fluid restriction, and urea; hiccups continued despite sodium correction to 138 mmol/L. Various treatments of hiccups including chlorpromazine, gabapentin, baclofen, metoclopramide, and antacids were trialed, but ultimately hiccups persisted for 11 days. Hiccups ceased following IV lorazepam administration, and sodium normalized thereafter. The patient was eventually able to discontinue all hyponatremia interventions post‐discharge with continued normal sodium (138–142 mmol/L) upon 2 months of follow‐up, and hiccups have remained in remission. Although other case reports have described hyponatremia causing hiccups whereby correcting the hyponatremia treated the hiccups, this is the first case demonstrating the reverse causal relationship. The anatomic findings of unusual GI anatomy (mesenteroaxial gastric malrotation with hiatal hernia) were assessed to be potential contributing factors to the refractory hiccups. The case also highlights the need to avoid anchoring to other common causes of SIADH, and supports a trial of lorazepam in refractory, severe hiccups.

## Graphical abstract

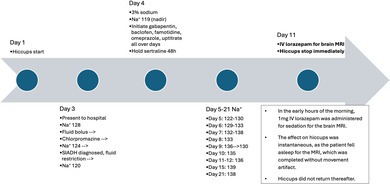

**Summary**

- Severe hiccups can induce the syndrome of inappropriate antidiuretic hormone, and intravenous lorazepam may be effective in refractory cases.
- Selective serotonin reuptake inhibitors should not be discontinued if the temporal association with hyponatremia is inconsistent.

## 1 Introduction

Hiccups are caused by myoclonic contractions of the diaphragm, comprised of a reflex arc which includes the phrenic and vagus nerves. Hiccups lasting > 48 h may indicate underlying pathology, with the most common etiologies being structural, vascular, and inflammatory, although the differential is broad. Some structural etiologies include those affecting the phrenic or vagus nerve, gastric irritants, and the medulla (demyelinating disease including neuromyelitis optica, vascular) [1]. Hyponatremia has been associated with hiccups in several case reports [2, 3, 4] for which treatment of hyponatremia has improved the hiccups; in other words, hyponatremia has seemed to trigger hiccups. Syndrome of inappropriate antidiuretic hormone (SIADH) etiology is broad, including central nervous system disorders, pulmonary disorders, malignancy (such as small cell lung cancer), and medications (including antidepressants), and may also include nausea and pain [5, 6]. This is a newly described case of severe hiccups which seemed to lead to hyponatremia (SIADH) as opposed to the reverse: treatment of the hyponatremia in this case did not improve hiccups. Rather, intravenous (IV) lorazepam was correlated with the resolution of hiccups, after failure of several other treatments. Hyponatremia resolved upon cessation of hiccups.

## 2 Case History/Examination

The patient is a 36‐year‐old with anxiety, depression, obsessive compulsive disorder, vasovagal syncope, and previously untreated gastroesophageal reflux (GERD) who presented with 48 h of spontaneous onset, severe, persistent hiccups causing significant discomfort and associated with nausea, vomiting, and intolerance of oral intake. Hiccups were described as clusters of five to seven repetitive, forceful diaphragmatic contractions occurring every 5–10 s, and these persisted during sleep. There were occasional periods of 1–3 h of quiescence approximately once per 24 h. The patient was alert and oriented, although uncomfortable, with frequent, loud hiccups, and had an otherwise unremarkable neurologic exam and a non‐tender abdomen.

## 3 Differential Diagnosis, Investigations, and Treatment

The patient was assessed upon initial presentation for suspected dehydration and provided a 1‐L bolus of lactated ringers (LR). Basic laboratory testing revealed hypokalemia (K^+^ 3.1 mmol/L; normal range 3.5–5.1 mmol/L), which was corrected, hyponatremia (Na^+^ 128 mmol/L), and hypochloremia (Cl^−^ 94 mmol/L; normal range 98–108 mmol/L). Hyponatremia was briefly attributed to dehydration for which the isotonic saline (LR) was provided (prioritized due to the patient's medical history of recurrent vasovagal syncope), but further reduction in serum sodium and corroborating urine studies revealed SIADH. The medication list includes sertraline 300 milligrams (mg) (dose stable for 1 year), which was held upon admission for 48 h due to concern that this medication may have caused SIADH: stopping the medication did not have an effect on sodium but caused withdrawal symptoms. Of note, as SIADH has a known association with nausea [5, 6], the set of nausea, vomiting, and poor oral intake persisted during symptom days #2–5, although hyponatremia persisted variably thereafter until symptom day #10. Serum sodium declined initially at a rate of 4 points per 12 h, and the nadir was 119 mmol/L (36 h after the first lab, and after treatment had already initiated). Sodium was corrected with 3% normal saline (NS); hiccups persisted despite sodium correction. A single dose of 25 mg chlorpromazine was given the first day of presentation, resulting in 5 h of hiccup cessation during sleep, but produced 24 h of intolerable akathisia, altered mental status, and urinary retention. Gabapentin, baclofen, and metoclopramide were then simultaneously administered with uptitrating doses, and omeprazole and famotidine were also initiated. A broad workup was performed as outlined in the below paragraph and table. The workup revealed abnormal stomach anatomy with gastric malrotation and a hiatal hernia. Hiccups persisted with high frequency for a total of 11 days, until administration of lorazepam (given prior to brain magnetic resonance imaging (MRI) testing), after which hiccup‐freedom was attained.

## 4 Summary of Investigations

**FIGURE 1:**
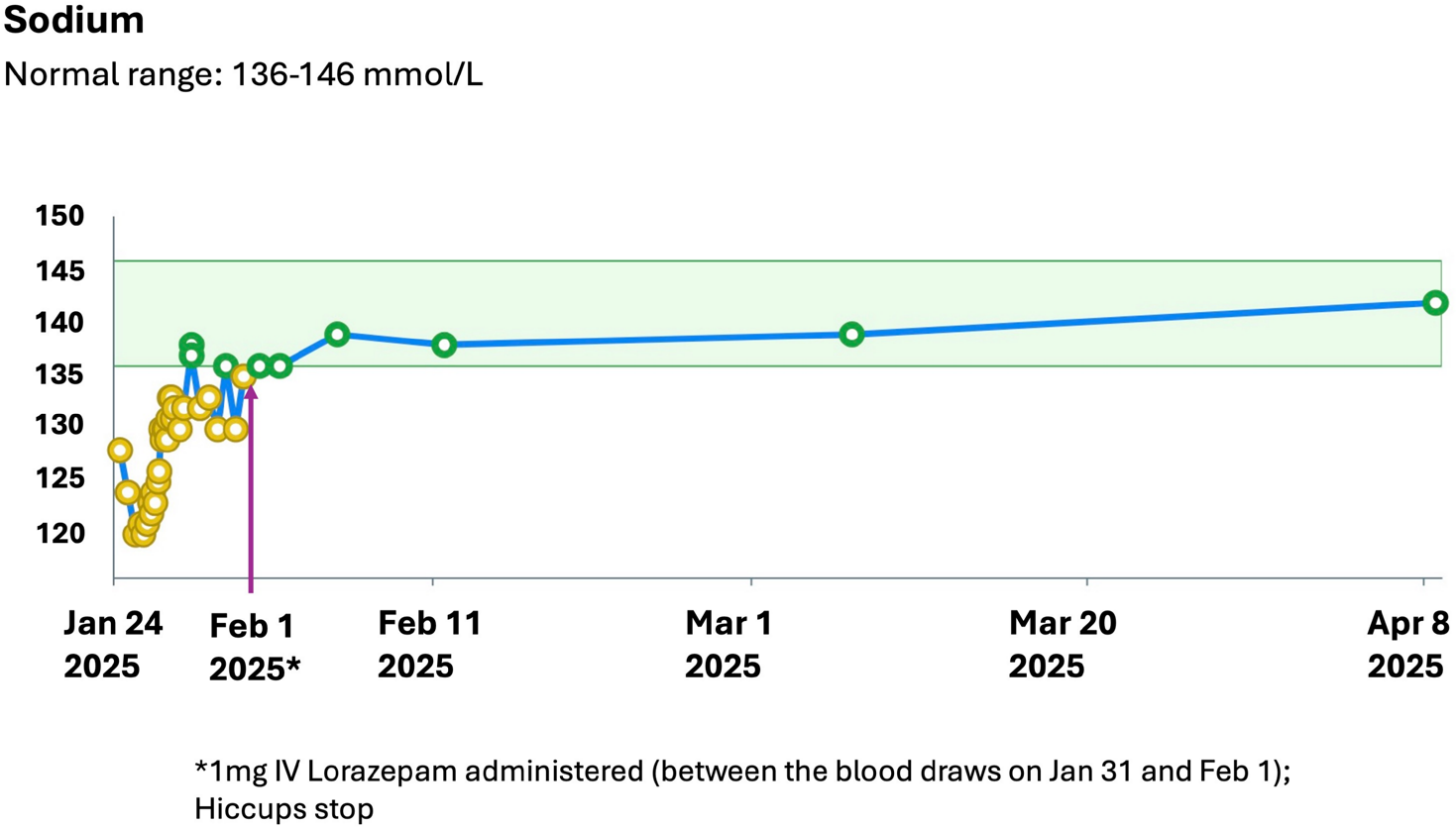
Sodium trends.

An electrocardiogram (EKG) and chest X‐ray (CXR) were normal. Head computed tomography (CT) and later brain MRI + cervical spine MRI were unremarkable. CT neck, chest, abdomen, and pelvis revealed a mild–moderate hiatal hernia, gaseous distension of the stomach and transverse colon, and elevated left hemi‐diaphragm. An upper gastrointestinal showed mesenteroaxial gastric malrotation “findings may be congenital in nature,” and no evidence of bowel obstruction. Sodium levels were trended starting hospital admission and through 2 months of follow‐up. The initial sodium was 128 mmol/L; nadir was 119 mmol/L at 36 h. The sodium corrected to normal for a period of time by symptom day #7 and persistently by symptom day #11 (Figure 1). Initial laboratory studies are summarized in Table 1.

**TABLE 1:** Initial laboratory studies.

Study	Value	Reference range
Sodium	**128**	136–146 mmol^a^/L
Potassium	**3.1**	3.5–5.1 mmol/L
Chloride	**94**	98–108 mmol/L
Urine sodium	87	N/A
Urine osmolality	344	300–1300 mOsm^b^/kg
Serum osmolality	**261**	269–298 mOsm/kg
Urine specific gravity	1.009	1.005–1.034
Urine pH	8.0	4.6–8.0
Thyroid‐stimulating hormone	1.87	0.3–5.5 mIU^c^/L
Morning cortisol	**26.2**	5.3–22.5 μg/dL
High sensitivity troponin	6	< 19 pg/mL
White blood cell count	8.5	4–10 thousand/μL
Hemoglobin	14.0	13.5–17 g/dL
Hematocrit	**37.5**	40%–50%
Platelet	**136**	150–400 thousand/μL
Aspartate aminotransferase	31	< 34 units/L
Alanine aminotransferase	**51**	10–49 units/L
Bilirubin, total	0.7	0.2–1.2 mg/dL
Urine drug screen 8	Negative	N/A
GCMS^d^	Negative	N/A
CNS demyelinating serum panel	Negative	N/A
Respiratory viral panel	Negative	N/A

*Note:* Bolded values are those outside of the laboratory reference range.

^a^ Millimole.

^b^ Milliosmole.

^c^ Milli‐international units.

^d^ Gas Chromatography Mass Spectroscopy.

## 5 Conclusion and Results (Outcome and Follow‐Up)

In management of hyponatremia, the patient was monitored in the intensive care unit; careful correction with 3% NS bolus and infusion allowed for improvement of the sodium (to 130 mmol/L by hospital day #3 = symptom day #5). This was followed by seven additional days of fluid‐restriction, salt tabs, and eventually urea, after which sodium normalized (with variability) to 138 mmol/L by symptom day #7 and ranged 138–142 mmol/L after discharge and by 2‐month follow‐up. There was some fluctuation of sodium values in the latter portion of the hospitalization despite stable urea and fluid restriction while the patient remained hiccupping, but values stabilized after hiccups stopped. The patient stopped salt tabs upon switching to urea and stopped fluid restriction at discharge.

Urea was continued prophylactically until 2‐month follow‐up; after cessation at that time, sodium remained normal. Sertraline was considered as a possible cause of SIADH, but this factor was ruled out after resuming sertraline within 48 h of cessation, without negative effect on the sodium.

Treatment with chlorpromazine was not tolerated, nor was the effect sustained throughout the drug's duration of action. The altered mental status correlated with both hyponatremia and chlorpromazine but was ultimately attributed to chlorpromazine because this resolved 24 h after the dose. Further, mental status normalized when the sodium was at its nadir (119 mmol/L).

Gabapentin (up to 400 mg three times per day (TID)), baclofen (up to 10 mg TID), metoclopramide (up to 15 mg TID), omeprazole (up to 40 mg two times per day (BID)), and famotidine (up to 20 mg BID) were added simultaneously, and thus it is not possible to separate the efficacy of an individual component; hiccups lessened in intensity but remained as frequent as onset throughout the hospitalization. Vomiting had improved significantly during the hospitalization and ceased by the sixth day of symptoms. Administration of 1 mg IV lorazepam on symptom day #11 (hospital day #9) was correlated with the resolution of hiccups and the patient was discharged the following day. Stomach malrotation, hiatal hernia, and GERD were likely risk factors for hiccups but were assessed by the surgeon to be non‐operative.

The patient was discharged on indefinite gabapentin, baclofen, omeprazole, famotidine, home sertraline, and brief metoclopramide, and continued urea until 2 months after discharge, with continued suppression of hiccups and normalized sodium.

## 6 Discussion

**FIGURE 2:**
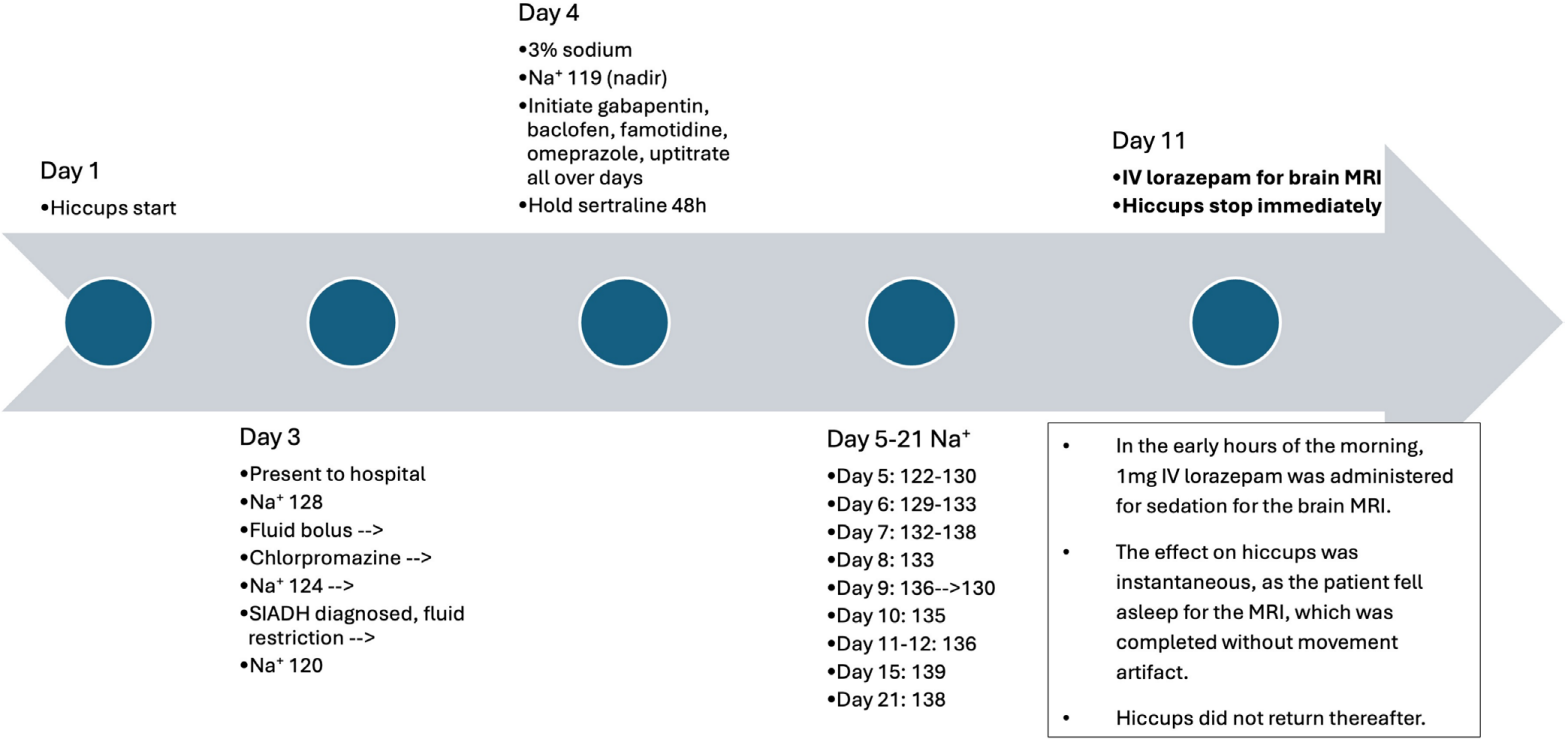
Timeline of hiccups, sodium, and treatments.

In the presenting case and in contrast to other case reports [2, 3, 4], the relationship between hiccups and hyponatremia (SIADH) appeared to be in the direction of hiccups causing SIADH: sodium was mildly low (128 mmol/L) upon admission after 48 h of hiccups, and correction of sodium to 138 mmol/L did not immediately treat hiccups (Figure 2). Further, other sources of SIADH were not identified as causative; although there was nausea and vomiting associated with these symptoms, they were short‐lived, yet fluctuating hyponatremia and hiccups remained ongoing. Additionally, the sodium normalized persistently through 2 months following treatment of the hiccups. Due to the lack of mechanistic studies, it cannot be conclusively determined that hiccups caused the hyponatremia; however, the temporal relationship between the hiccups and laboratory values is highly suggestive to this author in implicating the severe hiccups.

The reflex arc for hiccups involves afferents from the phrenic, vagus, and sympathetic nerves to the upper spinal cord and medulla, reticular formation, and hypothalamus—centrally mediated by dopaminergic and GABA‐ergic (gamma‐aminobutyric acid) neurotransmitters. The efferent branch is composed of the phrenic nerve, in addition to the accessory nerve to intercostal muscles, with subsequent glottic closure by the recurrent laryngeal branch of the vagus nerve [1, 7].

Many medications used in the treatment of hiccups affect the GABA pathway, not limited to gabapentin and baclofen [8]; it is therefore not surprising that the benzodiazepine, lorazepam, may exert similar benefit due to similar inhibitory effects. It should be noted that benzodiazepines such as midazolam have paradoxically been associated with the development of hiccups in some cases, including perioperatively [8]. However, one may hypothesize that there may be shared mechanisms between the pathophysiology of seizures and this aberrant reflex arc characterized by a form of myoclonus or diaphragmatic spasm. In this lens, the use of anti‐seizure medications is logical (this also incorporates gabapentin, valproic acid, carbamazepine, and phenytoin [1] which have been used in some cases). In this patient, it is unknown to what degree each of the medications may have improved the hiccups due to polypharmacy, but the temporal association between the administration of lorazepam and persistent cessation of hiccups was stark.

Limitations are several. In this patient, the initial etiology of hiccups remains unknown but is suspected to be multifactorial, such as related to the patient's abnormal stomach anatomy, untreated GERD, or other undetermined factors; a firm cause was not determined, and the patient seems to remain at risk for future recurrence. It is possible there may have been a bi‐directional relationship between hyponatremia perpetuating hiccups. Another significant limitation is the role of polypharmacy in treating the hiccups; the patient has started and uptitrated several traditional hiccup medications, and thus the independent role of lorazepam is not completely certain.

It is important not to anchor to selective serotonin reuptake inhibitors (SSRIs) as the cause of SIADH when there is data to the contrary. The patient's hyponatremia was acute, but the sertraline was a long‐term medication at a stable dose since 1 year prior. The patient developed significant withdrawal when the medication was held. The patient had described their experience with sertraline as “life changing” in the care of underlying mental health comorbidities and quality of life, and for this reason, the medication was re‐instated despite some etiologic uncertainty at the time of SIADH diagnosis.

Although rare, it is important to recognize the relationship between persistent hiccups and SIADH; while other case reports have cited hyponatremia (including SIADH) as a cause of hiccups, this case seems to demonstrate the reverse causal relationship.

In the case of sudden onset refractory hiccups, consideration may be given to a trial of IV lorazepam, which successfully and completely terminated this patient's severe hiccups.

## Author Contributions

**Lisa Matlen:** data curation, formal analysis, writing – original draft, writing – review and editing.

## Ethics Statement

The author's institution does not require ethical approval for reporting individual cases.

## Consent

A written informed consent was obtained from all the patient(s) to publish this report in accordance with the journal's patient consent policy.

## Conflicts of Interest

The author declares no conflicts of interest.

## Data Availability

All data underlying the results are available as part of the article and no additional source data are required.
